# Multilocus sequence analysis for the taxonomic updating and identification of the genus *Proteus* and reclassification of *Proteus* genospecies 5 O’Hara et al. 2000, *Proteus cibarius* Hyun et al. 2016 as later heterotypic synonyms of *Proteus terrae* Behrendt et al. 2015

**DOI:** 10.1186/s12866-020-01844-1

**Published:** 2020-06-10

**Authors:** Hang Dai, Binghuai Lu, Zhenpeng Li, Zhenzhou Huang, Hongyan Cai, Keyi Yu, Duochun Wang

**Affiliations:** 1 National Institute for Communicable Disease Control and Prevention, Chinese Center for Disease Control and Prevention (China CDC), State Key Laboratory of Infectious Disease Prevention and Control, Changbai Road 155, Changping, Beijing, 102206 China; 2 Center for Human Pathogen Collection, Chinese Center for Disease Control and Prevention, Changbai Road 155, Changping, Beijing, 102206 China; 3 Department of Pulmonary and Critical Care Medicine, China-Japan Friendship Hospital, Beijing, China

**Keywords:** *Proteus*, Multilocus sequence analysis, Taxonomy, Identification

## Abstract

**Background:**

Members of the genus *Proteus* are mostly opportunistic pathogens that cause a variety of infections in humans. The molecular evolutionary characteristics and genetic relationships among *Proteus* species have not been elucidated to date. In this study, we developed a multilocus sequence analysis (MLSA) approach based on five housekeeping genes (HKGs) to delineate phylogenetic relationships of species within the genus *Proteus*.

**Results:**

Of all 223 *Proteus* strains collected in the current study, the phylogenetic tree of five concatenated HKGs (*dnaJ, mdh, pyrC, recA* and *rpoD*) divided 223 strains into eleven clusters, which were representative of 11 species of *Proteus*. Meanwhile, the phylogenetic trees of the five individual HKGs also corresponded to that of the concatenated tree, except for *recA*, which clustered four strains at an independent cluster. The evaluation of inter- and intraspecies distances of HKG concatenation indicated that all interspecies distances were significantly different from intraspecies distances, which revealed that these HKG concatenations can be used as gene markers to distinguish different *Proteus* species. Further web-based DNA-DNA hybridization estimated by genome of type strains confirmed the validity of the MLSA, and each of eleven clusters was congruent with the most abundant *Proteus* species. In addition, we used the established MLSA method to identify the randomly collected *Proteus* and found that *P. mirabilis* is the most abundant species. However, the second most abundant species is *P. terrae* but not *P. vulgaris*. Combined with the genetic, genomic and phenotypic characteristics, these findings indicate that three species, *P. terrae*, *P. cibarius* and *Proteus* genospecies 5, should be regarded as heterotypic synonyms, and the species should be renamed *P. terrae*, while *Proteus* genospecies 5 has not been named to date.

**Conclusions:**

This study suggested that MLSA is a powerful method for the discrimination and classification of *Proteus* at the species level. The MLSA scheme provides a rapid and inexpensive means of identifying *Proteus* strains. The identification of *Proteus* species determined by the MLSA approach plays an important role in the clinical diagnosis and treatment of *Proteus* infection.

## Background

The genus *Proteus* belonging to the family *Enterobacteriaceae* is a motile gram-negative bacterium that survives in soil, water, and the intestinal tracts of mammals. Most members of the genus *Proteus* are opportunistic pathogens that cause a variety of infections in humans, including urinary tract infections [1], wounds, and respiratory tract, skin, eye, ear, nose, and throat infections [2].

The genus was first described by Hauser and was successively separated into two species, *Proteus mirabilis* and *Proteus vulgaris*, on the basis of the ability of these species to ferment maltose [2]. Strains of *P. vulgaris* comprised three biogroups based on three biochemical reactions, namely, indole production, salicin fermentation and aesculin hydrolysis. Biogroup 1 was characterized by being negative for those three reactions, named *P. penneri* [3]. By contrast, biogroup 2 was positive for the three reactions and retained the name *P. vulgaris*. Biogroup 3 was positive for indole production but negative for salicin fermentation and aesculin hydrolysis [4] and further separated into four groups by DNA-DNA hybridization, which were designated *Proteus* genospecies 3, 4, 5 and 6 [4]. Genospecies 3 can be distinguished from *Proteus* genospecies 4, 5 and 6 because it is negative for Jordan’s tartrate utilization and was named by the species of *P. hauseri*, while genospecies 4, 5 and 6 remained unnamed due to their undistinguishable phenotypic differentiation [4]. In addition, six newly defined species, i.e., *P. terrae* and *P. cibarius, P. alimentorum, P. columbae, P. faecis* and *P. cibi,* were proposed recently based on phylogenetic, phenotypic, chemotaxonomic and genotypic analyses [5–9]. Thus, the genus *Proteus* comprises ten validly published species and three unnamed genospecies to date (4, 5 and 6)*.*

Except for those six newly defined species, the classification of other *Proteus* species and genospecies was based on the difference in biochemical reactions and DNA-DNA hybridization, which were designed 19 years ago or even further in the past [4, 10]. In particular, the molecular evolutionary characteristics and genetic relationships among those *Proteus* phenospecies and genospecies have not been elucidated to date due to the absence of a molecular typing method in the *Proteus* genus. Multilocus sequence analysis (MLSA) based on several housekeeping genes (HKGs) has previously been successfully employed to delineate boundaries between closely related bacterial species, subspecies and component strains [11–13]. Partial sequences of protein-encoding genes have proven useful for species identification and as phylogenetic markers in the family *Enterobacteriaceae* [14, 15].

In the present study, we developed a five-gene MLSA approach to delineate genetic similarities and differences among *Proteus* species. We used this MLSA method to type the genotypic species of 223 *Proteus* strains that were identified by phenotypes. Our data indicate that MLSA is a powerful method for the discrimination, classification and phylogenetic analysis of *Proteus* at the species level; meanwhile, we revealed taxonomic relationships between phenotypic and genotypic species, specifically, modifying two phenotypic taxonomy using this MLSA method.

## Results

### MLSA of the five concatenated HKGs

**Fig. 1:**
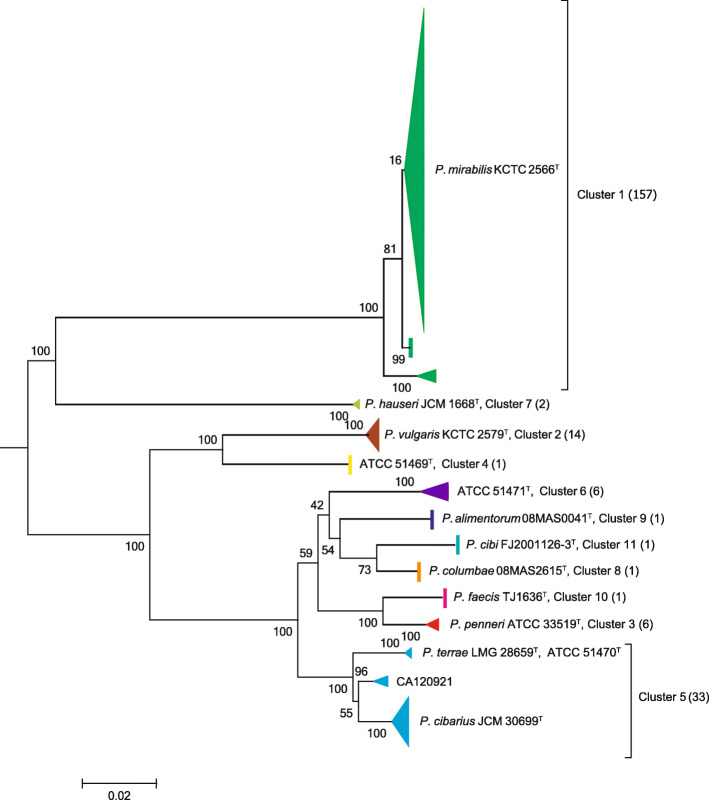
Phylogenetic reconstruction of *Proteus* strains based on concatenated *dnaJ, mdh, pyrC, recA* and *rpoD* gene sequences. The tree is based on 3157 nt of common sequences. Analysis was performed using the maximum-likelihood method. The scale bar indicates substitutions per site. The type strains were indicated in each cluster, if included. The strain number of each species is shown in parentheses

Of all 223 *Proteus* strains collected in this study, the phylogenetic tree of the concatenated 5 genes divided them into eleven clusters (Fig. 1), representing thirteen species. Among the clusters, ten contained one type strain of each. However, cluster 5 was comprised of three type strains, i.e., *Proteus* genospecies ATCC 51470^T^, *P. cibarius* JCM 30699^T^ and *P. terrae* LMG 28659^T^.

As expected, among the 223 *Proteus* strains, *P. mirabilis* (cluster 1) is the largest cluster (*n* = 157, 70.4%) distinctly separated from the others, and there are three subclusters within this cluster. Cluster 5 is the second largest among *Proteus* strains (*n* = 33, 14.8%) followed by *P. vulgaris* (cluster 2) (*n* = 14, 6.3%) and *P. penneri* (cluster 7) (*n* = 6, 2.7%).

### Identification of *Proteus* species by phylogenetic analysis of five individual genes

**Fig. 2:**
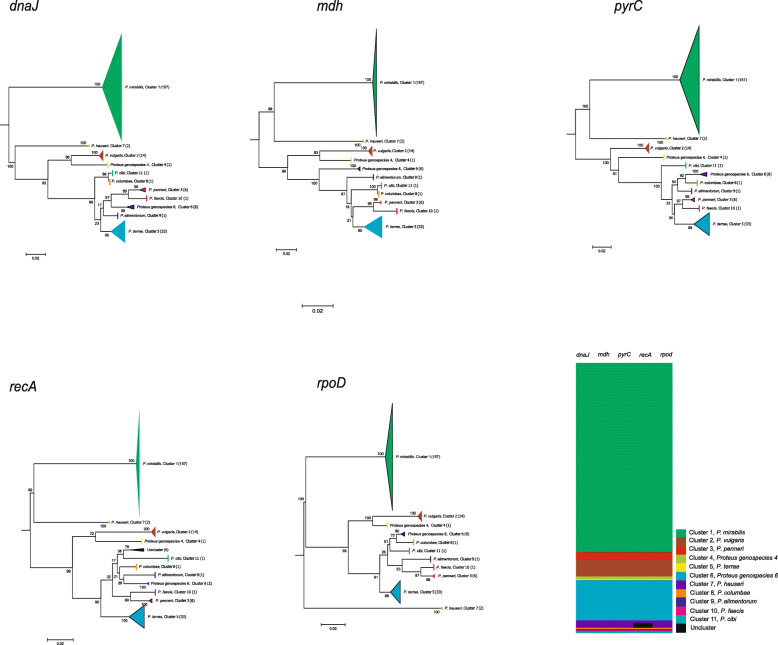
Phylogenetic reconstructions of *Proteus* strains based on five individual genes and their identification with five concatenated genes of species. The strain number of each species is shown in parentheses. The scale bar indicates substitutions per site

Phylogenetic trees based on five individual HKGs were also constructed (Fig. 2). Phylogenetic trees of the five HKGs (*dnaJ, mdh, pyrC, recA* and *rpoD*) can be divided into eleven clusters, representing eleven species and corresponding to that of the concatenated tree. Meanwhile, phylogenetic trees of four individual HKGs (*dnaJ, mdh, pyrC* and *rpoD*) were the same as that of the concatenated tree, both in numbers of species (cluster) and strain numbers within each species (cluster). There is one inconsistency between trees of *recA* and concatenated 5-gene: *recA* identified four strains as unclusters, whereas the four strains were identified by concatenated 5 genes, and the other four HKGs were identified as genospecies 6 (Fig. 2). The results showed that it is inaccurate to classify the species of *Proteus* by using a single housekeeping to reflect general gene phylogenetic tree and it only reflects the evolution by itself, which is caused by genetic recombination or specific selection. While the phylogenetic tree constructed by five concatenated HKGs can overcome the basis.

### Inter- and intraspecies distances of HKGs

**Fig. 3:**
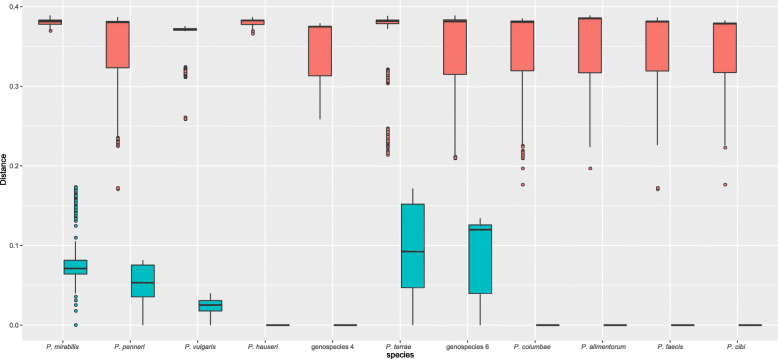
Intra- and interspecies distances of eleven species inferred by concatenated 5-gene MLSA. In each boxplot, from bottom to top: minimum, median and maximum. Inter: interspecies standard deviation (SD); Intra: intraspecies SD; *Proteus* genospecies 4, *P. columbae*, *P. alimentorum*, *P. faecis*, *P. cibi* including only one value and thus no intraspecies SD

The inter- and intraspecies distances of HKGs were summarized in a boxplot of the concatenated 5 genes (Fig. 3). All interspecies distances were clearly different from intraspecies distances. Among the interspecies boxplots, two species, *P. mirabilis,* and *P. hauseri,* indicated compacted distance ranges (both standard deviations, SD = 0.004), whereas the remaining nine species shared dispersive distance ranges (SD ranges from 0.024 to 0.065). On the other hand, among the intraspecies boxplots, *P. hauseri* possessed a compacted distance range (SD = 0.000) compared to that of five species (SD range from 0.012 to 0.058). Meanwhile, boxplots of the five individual genes (Figure S1) indicated the same trends of intra- and interspecies distance as that of the concatenated 5 genes, although there were small parts overlapping in species 5 and 6 of *pyrC*. The detailed genetic distance and median values of individual genes and the concatenated 5 genes are summarized in Table S1.

### Web-based DNA-DNA hybridizations among species

To confirm the correctness of strains among the eleven species, we used web-based DDH, such as dDDH and ANI, to detect their similarity values. Among the eleven species defined in this study, the dDDH and ANI values of the type/representative strains were 23.5–57.1% and 80.8–94.4% (Table 1), less than the proposed cutoff level for species delineation, i.e., 70 and 95%, respectively. Notably, among the three subclusters within cluster 5 (Fig. 1), either among the three published type strains (*Proteus* genospecies ATCC 51470^T^, *P. cibarius* JCM 30699^T^ and *P. terrae* LMG 28659^T^) or representative strain (CA142267) among the three subclusters, their dDDH and ANI values were more than the proposed cutoff level for species delineation. These results indicate that strains within cluster 5 actually belong to the same species.

**Table 1:** dDDH relatedness and ANI values among the eleven species

Strains ^a^		a		b		c		d		e		f		g		h		i		j		k		l		m		n	
Species		*P. mirabilis*		*P. penneri*		*P. vulgaris*		*P. hauseri*		*Proteus genospecies* 4		*Proteus genospecies* 5		*P. cibarius*		*P. terrae*		CA142267		*Proteus genospecies* 6		*P. columbae*		*P. alimentorum*		*P. faecis*		*P. cibi*	
DDH/ANI		DDH	ANI	DDH	ANI	DDH	ANI	DDH	ANI	DDH	ANI	DDH	ANI	DDH	ANI	DDH	ANI	DDH	ANI	DDH	ANI	DDH	ANI	DDH	ANI	DDH	ANI	DDH	ANI
Strains^a^	a	100.0	100.0																										
	b	24.7	82.1	100.0	100.0																								
	c	24.4	81.7	37.5	89.4	100.0	100.0																						
	d	23.5	80.8	25.8	83.3	25.1	82.6	100.0	100.0																				
	e	24.6	81.8	41.5	90.9	44.5	91.6	25.8	83.2	100.0	100.0																		
	f	24.3	81.8	44.6	91.7	37.7	89.6	25.8	83.1	40.0	90.2	100.0	100.0																
	g	25.1	82.6	44.5	91.7	37.5	89.6	25.7	83.2	39.9	90.2	72.3	96.9	100.0	100.0														
	h	24.1	81.5	44.6	91.8	37.5	89.5	25.7	83.3	39.9	90.2	93.1	99.2	71.2	96.7	100.0	100.0												
	i	24.4	82.0	44.9	91.8	37.6	89.7	25.8	83.1	39.8	90.3	74.4	97.1	72.3	96.8	72.6	96.8	100.0	100.0										
	j	25.0	82.4	44.8	91.7	36.0	89.0	26.1	83.4	37.8	89.5	47.3	92.3	47.1	92.5	47.3	92.4	48.3	92.8	100.0	100.0								
	k	24.8	81.9	46.3	92.1	36.8	89.1	26.0	83.4	37.9	89.6	48.9	92.7	48.3	92.6	48.8	93.0	49.8	93.0	57.1	94.4	100	100						
	l	24.9	82.0	46.3	92.1	36.4	89.0	25.9	83.3	37.8	89.6	48.7	92.8	48.1	92.7	48.5	92.7	49.2	92.3	52.4	93.7	53.9	93.9	100	100				
	m	24.1	81.6	52.7	93.7	35.8	88.7	25.7	83.1	36.5	89.1	45.9	91.9	45.5	91.9	45.9	92.0	46.6	92.1	47.1	92.2	48.6	92.7	48.5	92.6	100	100		
	n	24.6	81.9	45.3	92.0	35.5	88.3	25.8	83.0	36.5	89.0	45.8	92.0	45.5	92.0	45.9	92.0	46.6	92.3	49.2	92.0	51.4	93.3	50.5	93	49.5	92.9	100	100

^a^ Strain: a, *P. mirabilis* ATCC 29906^T^(GenBank accession no. ACLE00000000.1); b, *P. penneri* ATCC 33519^T^ (PHFJ00000000); c, *P. vulgaris* KCTC 2579^T^ (PHNN000000000); d, *P. hauseri* JCM 1668^T^ (PGWU00000000); e, *Proteus* genospecies 4 ATCC 51469^T^ (PENV00000000); f, *Proteus* genospecies 5 ATCC 51470^T^ (PENU00000000); g, *P. cibarius* JCM 30699^T^ (PGWT00000000); h, *P. terrae* LMG 28659^T^ (PENS00000000); i, CA142267; j, *Proteus* genospecies 6 ATCC 51471^T^ (PENT00000000); k, *P. columbae* 08MAS2615^T^ (NGVR00000000); l, *P. alimentorum* 08MAS0041^T^ (NBVR00000000); m, *P. faecis* TJ1636^T^ (PENZ00000000); n, *P. cibi* FJ2001126-3^T^ (PENW00000000)

Results were percentages based on Formula 2, calculate distances and DDH estimates with GGDC 2; ANI values were estimated using the web-based service ANI calculator (http://www.ezbiocloud.net/tools/ani)

### Reclassification of *Proteus* genospecies 5 and *P. cibarius* to *P. terrae*

Since either MLSA of the five concatenated HKGs or phylogenetic analysis of five individual genes indicated that three type strains, i.e., *Proteus* genospecies ATCC 51470^T^, *P. cibarius* JCM 30699^T^ and *P. terrae* LMG 28659^T^, fell into one cluster (cluster 5 in Fig. 1), further web-based DNA-DNA hybridizations, such as dDDH and ANI, confirmed that among the three subclusters within cluster 5, either among the three type strains or representative strain (CA142267) among the three subclusters, their dDDH and ANI values were higher than the proposed cutoff level for species delineation (70% for dDDH and 95% for ANI, Table 1). The genomic analysis provided evidence that strains within cluster 5 actually belonged to the same species.

Further phenotypic characteristics were detected among type strains of *Proteus* genospecies 5, *P. cibarius* and *P. terrae*, and slight distinctive properties were observed (Table 2). Only minor differences were obtained between the type strains of the three species, including growth at the optimum temperature, growth range in NaCl and pH, utilization of DNase, lipase and citric acid, and DNA G + C content. Combined with the genetic, genomic and phenotypic characteristics, three species, *P. terrae* reported by Behrendt et al. 2015, *P. cibarius* reported by Hyun et al. 2016 and *Proteus* genospecies 5 reported by O’Hara et al. 2000, should be regarded as the heterotypic synonyms of *Proteus terrae* reported by Behrendt et al. 2015.

**Table 2:** Distinctive phenotypic characteristics among the type strains *P. terrae*, *P. cibarius* and *Proteus* genospecies 5^a^

Characteristic	*P. terrae*	*P. cibarius*	*Proteus* genomospecies 5
Growth in optimum temperature (°C)	37	35	37
Growth range in NaCl (%,w/v)	0–15	0–12	0–15
Growth range in pH	4–9	4–9	4–9
DNase (25 °C) (3 days)	+	+	+
Lipase (olive oil) (7 days)	–	+	–
CIT	–	–	+
DNA G + C content (mol %)	37.9	37.8	37.8

^a^Species strain: *P. terrae* LMG 28659^T^; *P. cibarius* JCM 30699^T^; *Proteus* genospecies 5 ATCC 51470^T^

## Discussion

MLSA has been used for classification at the species level in numerous *Enterobacteriaceae* [14–21]. MLSA has the advantage of being more convenient and more conducive to popularization in the primary research institution than the whole genome sequencing method. Normally, four to seven HKGs were selected for MLSA to determine phylogenetic relationships. It has been recommended that researchers use sequence data from more than one gene to reduce the possibility of ambiguities caused by genetic recombination or specific selection. MLSA is increasingly applied to obtain a higher resolution power between species within a genus and provides a perspective for the genotypic taxonomic analyses of genus *Proteus* [22]. In this study, the five housekeeping genes (*dnaJ, mdh, pyrC, recA,* and *rpoD*) contain high conservative sequence and high variable sequence, which are considered to have a slow and constant rate of evolution and resolution in the distinction of species level. When amplified by PCR of 223 tested *Proteus* strains collected, the five HKGs sequence data were deposited to NCBI GenBank and have a good corresponding relationship of consistency among different species. Thus, we established the MLSA method with the five genes for taxonomic analysis of the *Proteus* genus. Our MLSA-based approach can be used to effectively discriminate *Proteus* sp. and enable the delineation of species boundaries with high confidence. To the best of our knowledge, this report describes the first MLSA method to classify the genus *Proteus* at the species level.

Our MLSA method divided all 223 *Proteus* strains into eleven clusters, representative of eleven species, which is inconsistent with the thirteen *Proteus species* in the current literature in subsequent studies; we confirmed that there are eleven *Proteus* species by using MLSA. Among the eleven species, *P. mirabilis* was the majority species collected in this study, which agrees with numerous reports of the *Proteus* genus classified by phenotypic methods, and the most common cause of the intentional disease is *Proteus mirabilis* [2]. However, even all *P. mirabilis* isolates were phenotypic with the same distinguishing biochemical features, i.e., positive for ornithine decarboxylase but negative for sucrose and maltose only. Species *P. mirabilis* can be further divided into three dominant subclusters, representing three subtypes that have demonstrated no biochemical difference or genetic difference. In contrast, species *P. vulgaris* was the most conserved cluster among the eleven species and exhibited one of the minimum intraspecies distances of HKGs (Fig. 2). Traditional biochemical identification *P. vulgaris* includes biogroup 2 and biogroup 3. By using MLSA, the *P. vulgaris* includes biogroup 2. Interestingly, *P. hauseri* was phylogenetically more closely to *P. mirabilis* than any other species (Fig. 1), although *P. hauseri* was previously classified to biogroup 3 of *P. vulgaris* [4]. MLSA as an alternative method for the whole genome sequence analysis is more accurate than biochemical identification of *Proteus* species. Cluster 5 included three subclusters, and the web-based DDH and ANI values indicated that strains within the cluster (including three type strains, *Proteus* genospecies ATCC 51470^T^, *P. cibarius* JCM 30699^T^ and *P. terrae* LMG 28659^T^) actually belong to the same species. *P. cibarius* and *P. terrae* were defined as new species of the genus *Proteus*, possibly because both studies excluded type strain of *Proteus* genospecies 5 (such as ATCC 51470^T^) [4]. Meanwhile, papers of the two species were accepted for publication recently (2015 and 2016) at different journals [5, 6] to ensure that they did not cite each other. We also emended three subclusters of cluster 5 into *Proteus terrae.*

*Proteus* is the most common opportunistic pathogen, of which *P. mirabilis* and *P. vulgaris* have long been considered the two most common species [2, 23, 24]. Clinically, different treatment schemes may be adopted according to the most abundant species of *Proteus* [25, 26]. In this study, we used the established MLSA method to identify the randomly collected *Proteus* and found that *P. mirabilis* is the most common genospecies of *Proteus*. However, the second most common is *P. terrae* but not *P. vulgaris*, and this result is notably different from that of clinical phenotype identification [2]. The reason for this finding is that in the clinic, strains of *Proteus* genospecies 4, 5 and 6 have long been identified as *P. vulgaris* by phenotypic biochemical reactions [4]; meanwhile, the result of this study indicates that *Proteus* genospecies 5 accounts for a large proportion (Fig. 1). Moreover, *P. penneri* and *P. hauseri* are initially classified as different biogroups of *P. vulgaris* [4]. Because accurate identification at the species level is of great significance for the clinical treatment of *Proteus* infection, MLSA-based identification should be suggested in the classification of the *Proteus* genus.

### Emended description of *Proteus terrae* Behrendt et al. 2015

*Proteus terrae* (ter’rae. L. gen. n. *terrae* of the soil).

*Proteus terrae* are gram-negative, straight-rod-shaped, motile bacteria that occur singly or in pairs [6]. Cells are facultatively anaerobic and swarm with periodic cycles when cultured on a 1.5% agar nutrient medium. The range of growth temperature is from 10 °C to 45 °C, and the optimum temperature is 37 °C. The range of salt tolerance is from 0 to 15%, and the optimum NaCl is 1%. The API 20E strain is positive for indole production and maltose and negative for ornithine decarboxylase, citrate utilization, and amygdalin. Based on the API, the 50CH strain is positive for L-rhamnose and sucrose and negative for arbutin, aesculin, and salicin. The strain type of *Proteus terrae* is LMG 28659^T^ (=DSM 29910^T^ = N5/678).

## Conclusions

This study suggested that MLSA is a powerful method for the discrimination and classification of *Proteus* at the species level. The MLSA scheme provides a rapid and inexpensive means of identifying *Proteus* strains. The identification of *Proteus* species determined by the MLSA approach plays an important role in the clinical diagnosis and treatment of *Proteus* infection. First, in comparison with the phenotypic biochemical classification (species) method, all tested strains can be divided into eleven clusters (genospecies) by the MLSA method, representing eleven species of *Proteus*. Second, our study revealed the phenospecies of strains composed of different genotypes at different phylogenetic scales. Third, our MLSA method proposed the emendation of the description of the genus *Proteus*: *P. terrae*, *P. cibarius* and *Proteus* genospecies 5, should be regarded as heterotypic synonyms, and the species should be renamed *P. terrae*.

## Methods

### Definition of species, phenospecies and genospecies in this study

To classify the biotype and genotype of *Proteus* isolates, we referred to the literature. We designated “phenospecies” as species identified by phenotypic traits, such as biochemical reactions; “genospecies” refer to genotype identified by MLSA of this study. To maintain consistency, genospecies 3, 4, 5 and 6 are equal to genomospecies 3, 4, 5 and 6 reported by O’Hara [4].

### Bacterial isolates and biochemical identification

A total of 223 *Proteus* strains were analyzed in this study. Among these strains, 210 were collected and isolated from seven provinces in China from 2002 to 2015. Specifically, 181 strains were isolated from clinical samples (feces of diarrhea patients and patients with nosocomial infections, such as blood, urine and wounds), and 29 strains were isolated from food. The 210 strains were identified as *Proteus* species by biochemical tests (API 20E). Meanwhile, type strains of ten *Proteus* species and three genospecies were also obtained and used for MLSA and biochemical tests, i.e., *P. mirabilis* KCTC 2566^T^, *P. vulgaris* KCTC 2579^T^, *P. penneri* ATCC 33519^T^, *P. hauseri* JCM 1668^T^, *P. cibarius* JCM 30699^T^, *P. terrae* LMG 28659^T^, *P. columbae* 08MAS2615^T^, *P. alimentorum* 08MAS0041^T^, *P. faecis* TJ1636^T^, *P. cibi* FJ2001126-3^T^, genospecies 4 (ATCC 51469^T^), 5 (ATCC 51470^T^) and 6 (ATCC 51471^T^). Separate biochemical tests (salicin fermentation, aesculin hydrolysis, DNase, lipase, acetate utilization and tartrate) were assessed using agents (Guangdong HuanKai Microbial Technology Co., Ltd.) in accordance with the manufacturer’s instructions. Experiments that did not yield clear results were performed in triplicate.

### DNA extraction, PCR amplification and sequencing

The genomic DNA from *Proteus* strains was extracted using a genomic DNA purification kit (Tiangen Biotech, Beijing, China) in accordance with the manufacturer’s instructions. Extracted DNA was dissolved in TE buffer and stored at − 20 °C until use as PCR templates. Five candidate HKGs were used for MLSA analysis, i.e., *dnaJ, mdh, pyrC, recA* and *rpoD.* The primer sets were designed and are listed in Table 3. For PCR amplification, each reaction was performed in a final volume of 50 μl containing 25 μl of 2× Taq PCR MasterMix (Tiangen Biotech, Beijing, China), 1.5 μl 10 μM of each forward and reverse primer, 2 μl DNA template, and 20 μl ddH_2_O. The reaction mixture was subjected to denaturation at 95 °C for 5 min followed by 30 cycles of denaturation at 95 °C for 30 s, annealing at 52 to 55 °C for 30 s and extension at 72 °C for 1 min/kb. An extension step of 10 min at 72 °C was performed following the last cycle to ensure full-length synthesis of the fragment. All PCR products of the five HKGs were commercially directly sequenced in both directions (TsingKe Biological Technology, Beijing, China). The forward and reverse sequences of each housekeeping gene were assembled by using DNASTAR’s lasergene sequence analysis software.

**Table 3:** PCR primers used in this study

Primer	Nucleotide sequence (5′ to 3′)	Amplicon size (bp)	Tm
dnaJ-F	CRATGAAATATCACCCAGAYCG	790	55 °C
dnaJ-R	ACACGRCCATCMAGWGTT		
mdh-F	GCAAAGAAACGGGCATRTT	769	55 °C
mdh-R	CRGGTGGTATTGGTCAGG		
pyrC-F	TGATTGGCATGTTCACTT	745	52 °C
pyrC-R	GATTCTTTGCGATGTTGT		
recA-F	CTRTACCAWGCACCMGCTT	807	52 °C
recA-R	AGGKTCTATCATGCGTCT		
rpoD-F	CGGGAAGGTGAAATTGAT	775	52 °C
rpoD-R	CGATAGACATACGACGGT		

### MLSA analysis

Phylogenetic trees were constructed by MLSA of the concatenated sequence of five HKG fragments (*dnaJ-mdh-pyrC-recA*-*rpoD,* 3157 bp) and the five individual HKGs. The total lengths of the alignments used were 629 bp (*dnaJ*), 635 bp (*mdh*), 647 bp (*pyrC*), 701 bp (*recA*) and 545 bp (*rpoD*). Comparison analyses of the sequences were conducted with BioEdit software (Ibis Biosciences, Carlsbad, CA, USA). ClustalW was used to perform multiple alignments of the nucleotide sequences. The phylogenetic analysis was performed using MEGA 7.0 for the maximum-likelihood (ML) method. In the ML method, the General Time Reversible model which is very extensive model in constructing the phylogenetic tree was selected, and the rate matrix, the base frequencies, the invariable site proportion and the gamma distribution were determined via likelihood. Phylogenetic tree branch support estimation and 1000 replications were calculated to obtain the bootstrap values.

### Intra- and interspecies phylogenetic distance of HKGs

Intraspecies phylogenetic distance was defined as the phylogenetic distance within the strains from the same species, and interspecies phylogenetic distance was defined as the phylogenetic distance of strains from a species with strains from other species. The phylogenetic distance between strains was calculated using MEGA 7.0 with the Kimura 2 parameter model which is the default model to calculate the distance. The minimum, median, and maximum intra- and interspecies values for each species were calculated. Variance of compacted or dispersive distance of species analyzed using Fisher’s exact test.

### Genomic relatedness among isolates of different species

The genomic relatedness among isolates of different species was further evaluated by web-based DNA-DNA hybridizations (DDH), such as in silico DDH (dDDH) and average nucleotide identity (ANI) to detect their similarity values [27, 28]. dDDH values were determined using the genome-to-genome distance calculator (GGDC) web server (http://ggdc.dsmz.de/), and ANI values were measured by the EZ BioCloud platform (http://www.ezbiocloud.net/tools/ani), with similarity values of 70 and 95% as the standard threshold for species boundaries, i.e., two isolates represented different species when their dDDH and ANI values were below the 70 and 95% thresholds, respectively [27, 28]. Except for *P. mirabilis* ATCC 29906^T^, all of the other test *Proteus* strains’ whole genome sequences were sequenced by our group, and these data were deposited into the NCBI database. The GenBank accession numbers are listed below in Table 1 [7–9].

## Supplementary information

**Additional file 1: Figure S1.** Intra- and inter-species distances of eleven species infer by five individual genes. In each boxplot, from bottom to top: minimum, median and maximum. **Table S1.** Intra- and inter-species genetic distance median values and ranges of concatenated 5-gene and five individual genes.

## Data Availability

The nucleotide sequences of five HKGs are deposited in the GenBank nucleotide sequence database under the accession numbers *dnaJ*: MG492023-MG492065, MG492068-MG492222, MG492228, MG492230-MG492232, MG492234-MG492250; *mdh*: MG492251-MG492295, MG492298-MG492452, MG492458, MG492460, MG492462-MG492478; *pyrC*: MG492479-MG492515, MG492518-MG492672, MG492678, MG492680, MG492682-MG492706; *recA*: MG492707-MG492733, MG492735, MG492737-MG492738, MG492743-MG492897, MG492900-MG492934; *rpoD*: MG492935-MG492959, MG492961, MG492963, MG492969-MG493125, MG493128-MG493162.

## Abbreviations

MLSA: Multilocus sequence analysis
HKGs: Housekeeping genes
ANI: Average nucleotide identity
dDDH: Digital DNA-DNA hybridizations
