# Supplementary material for: Multilocus sequence analysis for the taxonomic updating and identification of the genus Proteus and reclassification of Proteus genospecies 5 O’Hara et al. 2000, Proteus cibarius Hyun et al. 2016 as later heterotypic synonyms of Proteus terrae Behrendt et al. 2015
Source: BMC Microbiol. 2020 Jun 10;20:152. doi: 10.1186/s12866-020-01844-1 (PMC7288399; doi:10.1186/s12866-020-01844-1)
Supplement: Supplementary file 1 — Additional file 1: Figure S1. Intra- and inter-species distances of eleven species infer by five individual genes. In each boxplot, from bottom to top: minimum, median and maximum. Table S1. Intra- and inter-species genetic distance median values and ranges of concatenated 5-gene and five individual genes. [file 12866_2020_1844_MOESM1_ESM.pdf]

BMC Microbiology

A multilocus sequence analysis for the taxonomic update and identification of the genus *Proteus*

Hang Dai[1,2], Binghuai Lu[3], Zhenpeng Li[1], Zhenzhou Huang[1,2], Hongyan Cai[1,2], Keyi Yu[1,2], Duochun Wang[1,2]*

[1]National Institute for Communicable Disease Control and Prevention, Chinese Center for Disease Control and Prevention (China CDC), State Key Laboratory of Infectious Disease Prevention and Control, Beijing, 102206, China.

[2] Center for Human Pathogen Collection, Chinese Center for Disease Control and Prevention, Beijing, China.

[3] Department of Pulmonary and Critical Care Medicine, China-Japan Friendship Hospital, Beijing, China.

Running title: Multilocus sequence analysis of genus *Proteus.*

*Correspondence Author: Duochun Wang, wangduochun@icdc.cn. Changbai Road 155, Changping, Beijing, 102206, China.

Supplementary figure legends

dnaJ

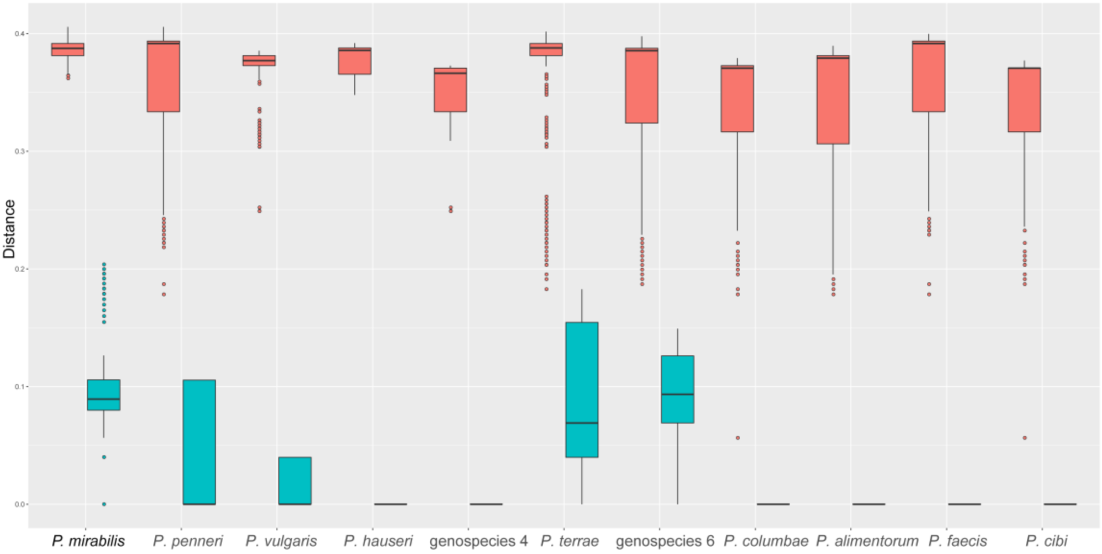

mdh

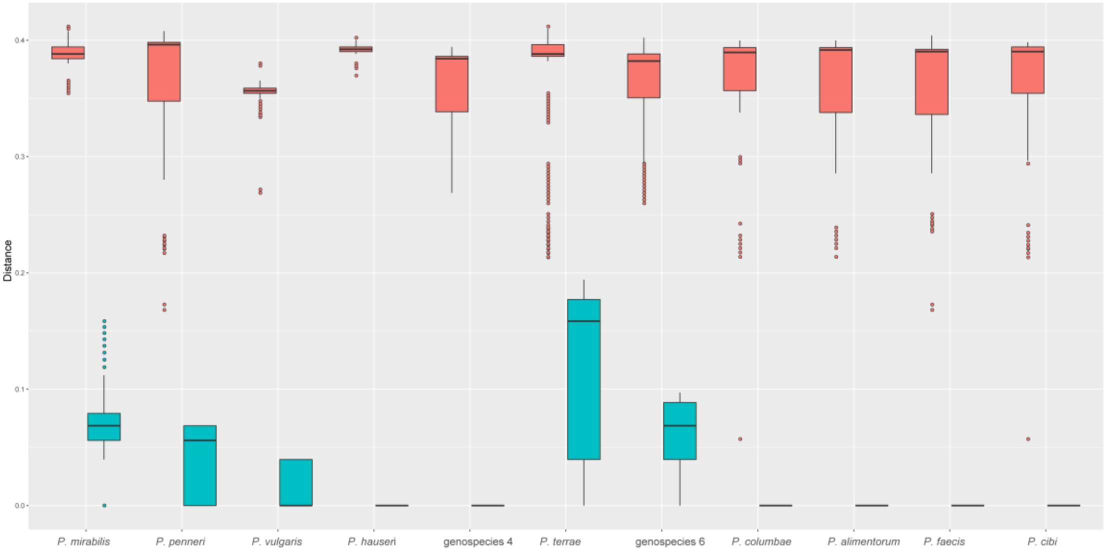

pyrC

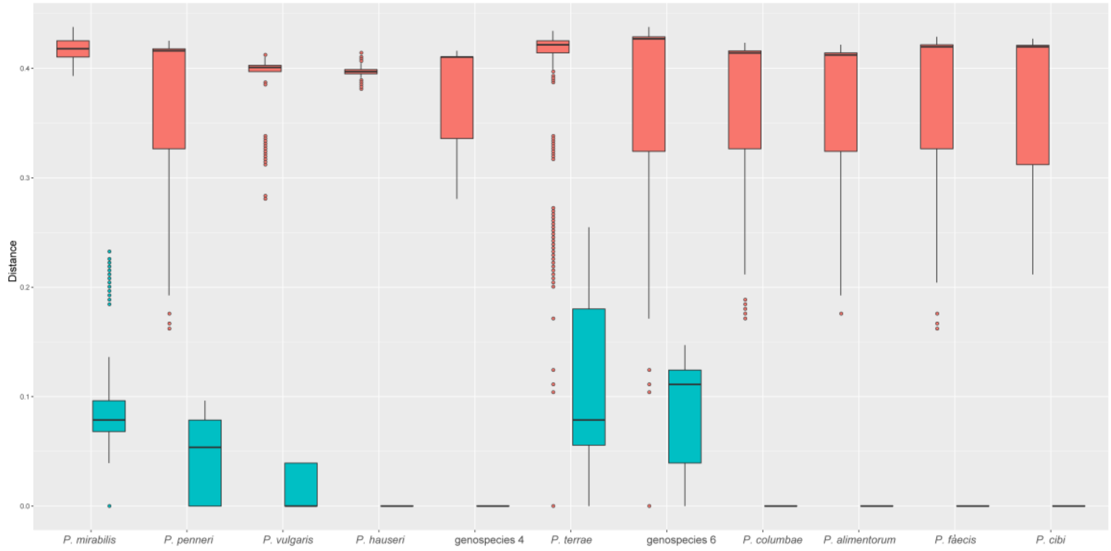
Distance
0.4
0.3
0.2
0.1
0.0
P. mirabilis
P. penneri
P. vulgaris
P. hauseri
genospecies 4
P. terrae
genospecies 6
P. columbae
P. alimentorum
P. fåecis
P. cibi

recA

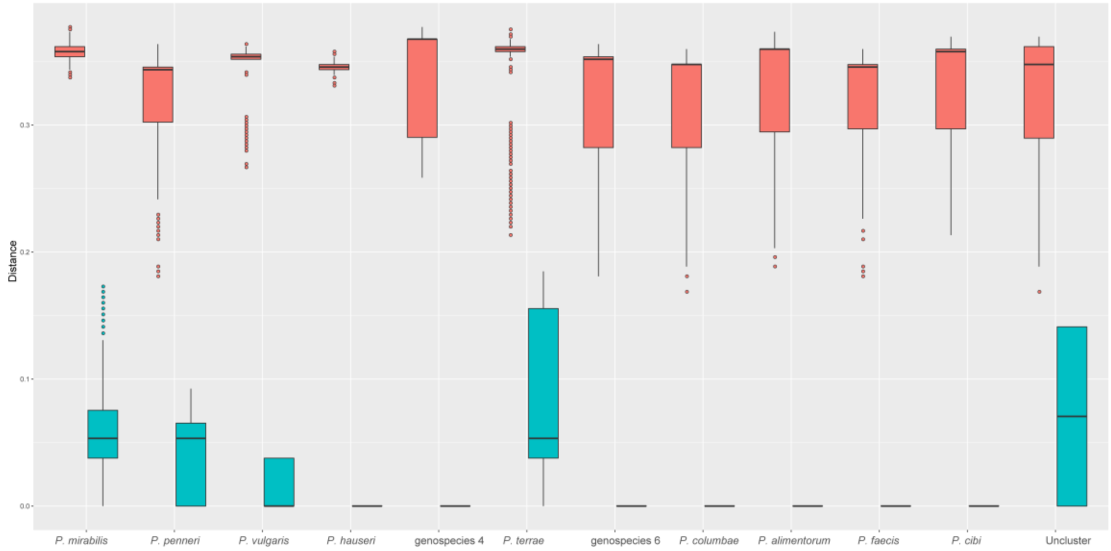
Distance
0.3
0.2
0.1
0.0
P. mirabilis
P. penneri
P. vulgaris
P. hauseri
genospecies 4
P. terrae
genospecies 6
P. columbae
P. alimentorum
P. faecis
P. cibi
Uncluster

rpoD

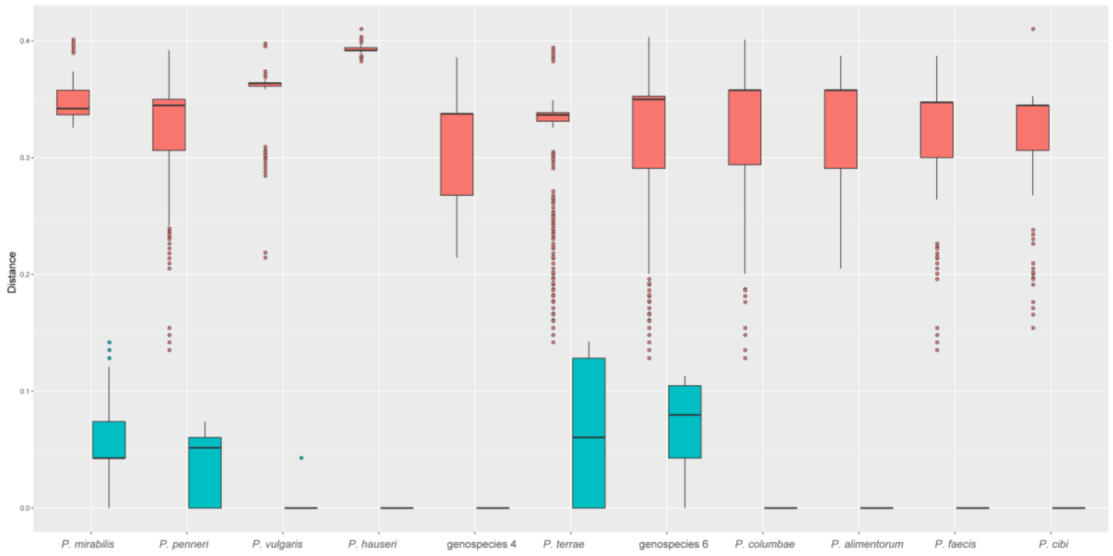

Figure S1. Intra- and inter-species distances of eleven species infer by five individual genes. In each boxplot, from bottom to top: minimum, median and maximum.

Table S1. Intra- and inter-species genetic distance median values and ranges of concatenated 5-gene and five individual genes

Gene	Intra/inter species	Species (median values [ranges])																							
		P. mirabilis		*P. penneri*		*P. vulgaris*		*P. hauseri*		genospecies 4		*P. terrae*		genospecies 6		*P. columbae*		*P. alimentorum*		*P. faecis*		*P. cibi*		uncluster	
Concatenated 5-gene	intra	0.07	[0, 0.17]	0.05	[0, 0.08]	0.03	[0, 0.04]	0	0	0	0	0.09	[0, 0.17]	0.12	[0, 0.13]	0	0	0	0	0	0	0	0	-	-
	inter	0.38	[0.37, 0.39]	0.38	[0.17, 0.39]	0.37	[0.26, 0.38]	0.38	[0.37, 0.39]	0.37	[0.26, 0.38]	0.38	[0.21, 0.39]	0.38	[0.21, 0.39]	0.38	[0.18,0.39]	0.39	[0.20,0.39]	0.38	[0.17,0.39]	0.38	[0.18,0.38]	-	-
dnaJ	intra	0.09	[0, 0.20]	0	[0, 0.11]	0	[0, 0.04]	0	0	0	0	0.07	[0, 0.18]	0.09	[0, 0.15]	0	0	0	0	0	0	0	0	-	-
	inter	0.39	[0.36, 0.41]	0.39	[0.18, 0.41]	0.38	[0.25, 0.39]	0.39	[0.35, 0.39]	0.37	[0.25, 0.37]	0.39	[0.18, 0.40]	0.39	[0.19, 0.40]	0.37	[0.06,0.38]	0.38	[0.18,0.40]	0.39	[0.18,0.40]	0.37	[0.07,0.38]	-	-
mdh	intra	0.07	[0, 0.16]	0.06	[0, 0.07]	0	[0,0.04]	0	0	0	0	0.16	[0, 0.19]	0.07	[0,0.10]	0	0	0	0	0	0	0	0	-	-
	inter	0.39	[0.35, 0.41]	0.4	[0.17, 0.41]	0.36	[0.27, 0.38]	0.39	[0.37, 0.40]	0.38	[0.27, 0.39]	0.39	[0.21, 0.41]	0.38	[0.26, 0.40]	0.39	[0.06,0.40]	0.39	[0.21,0.40]	0.39	[0.17,0.40]	0.39	[0.06,0.40]	-	-
pyrC	intra	0.08	[0, 0.23]	0.05	[0, 0.10]	0	[0, 0.04]	0	0	0	0	0.08	[0, 0.25]	0.11	[0, 0.15]	0	0	0	0	0	0	0	0	-	-
	inter	0.42	[0.39, 0.44]	0.42	[0.16, 0.43]	0.4	[0.28, 0.41]	0.4	[0.38, 0.41]	0.41	[0.28,0.42]	0.42	[0, 0.43]	0.43	[0, 0.44]	0.41	[0.17,0.42]	0.41	[0.18,0.42]	0.42	[0.16,0.43]	0.42	[0.21,0.43]	-	-
recA	intra	0.07	[0, 0.14]	0.05	[0, 0.09]	0	[0,0.04]	0	0	0	0	0.05	[0, 0.18]	0	0	0	0	0	0	0	0	0	0	0.07*	[0,0.14]*
	inter	0.35	[0.17,0.37]	0.34	[0.18, 0.36]	0.35	[0.27, 0.37]	0.35	[0.33, 0.36]	0.37	[0.26, 0.38]	0.36	[0.21, 0.38]	0.35	[0.18, 0.36]	0.35	[0.17,0.36]	0.36	[0.19,0.37]	0.35	[0.18,0.36]	0.36	[0.21,0.37]	0.35*	[0.17,0.37]*
rpoD	intra	0.04	[0, 0.14]	0.05	[0, 0.07]	0	[0, 0.04]	0	0	0	0	0.06	[0, 0.14]	0.08	[0, 0.11]	0	0	0	0	0	0	0	0	-	-
	inter	0.34	[0.33, 0.40]	0.34	[0.14, 0.39]	0.36	[0.21, 0.40]	0.39	[0.38, 0.41]	0.34	[0.21, 0.39]	0.34	[0.14, 0.40]	0.35	[0.13, 0.40]	0.36	[0.13,0.40]	0.36	[0.21,0.39]	0.35	[0.14,0.39]	0.34	[0.15,0.41]	-	-

*: The median values and ranges of uncluster in *recA* genes
